# Effects of Ultrasound-Assisted Extraction on Yield, Physicochemical Properties, and Structural Characterization of *Rosa laevigata* Polysaccharides: A Comparative Analysis with Five Conventional Methods

**DOI:** 10.3390/foods14244275

**Published:** 2025-12-12

**Authors:** Yunxin Shi, Xiangying Zuo, Ziyu Han, Xuqin Song, Jian Yang, Ya Song

**Affiliations:** 1 School of Food Engineering, Moutai Institute, Renhuai 564507, China; 2 College of Animal Science, Guizhou University, Guiyang 550025, China; 3 Key Laboratory of Genetic Breeding and Reproduction of Highland Mountain Animals of Ministry of Education, Guizhou University, Guiyang 550025, China; 4 Guizhou Engineering Research Center for Health-Functional Liquor Brewing Technology, Moutai Institute, Renhuai 564507, China

**Keywords:** ultrasound-assisted extraction (UAE), *Rosa laevigata* polysaccharides, yield, physicochemical properties, structural characterization

## Abstract

This study systematically compared ultrasound-assisted extraction (UAE) with five other methods (hot water extraction (HWE), microwave-assisted extraction (MAE), acid extraction (FTACP), alkali extraction (FTAIP), and enzyme-assisted extraction (EAE)) for their effects on the yield, physicochemical properties, and bioactivities of *Rosa laevigata* Michx. polysaccharides. The results demonstrated UAE’s superior performance: it achieved a higher polysaccharide yield (31.27%) than HWE, FTACP, and FTAIP, approaching that of MAE and EAE. SEM observation revealed that UAE-derived polysaccharides exhibited a uniform porous network with smooth surfaces and excellent dispersibility, outperforming the irregular aggregates or structural loosening observed in other methods. Notably, UAE polysaccharides showed remarkable cholesterol-binding capacity (31.18 mg/g) and FRAP reducing power (0.0423 mmol/g), which highlights their potential for functional food applications. Structural analyses (FT-IR, XRD, TGA) confirmed that UAE better preserved the native conformation and thermal stability of polysaccharides, whereas chemical (FTACP/FTAIP) and high-temperature (MAE) methods induced molecular degradation. In conclusion, UAE, as an eco-friendly and low-denaturation technique, offers an optimal strategy for the high-value utilization of *R. laevigata* polysaccharides.

## 1. Introduction

*Rosa laevigata* Michx. belongs to the genus *Rosa* of the Rosaceae family [1]. Its roots, stems, leaves, flowers, and fruits are all for both medicinal and edible purposes [2]. The *Compendium of Materia Medica* records that *R. laevigata* Michx. primarily treats spleen deficiency diarrhea, excessive urination, and astringe essence; long-term use can enhance cold tolerance, lighten the body, and enrich blood and essence [3]. It contains abundant nutritional compounds such as unsaturated fatty acids and essential amino acids, as well as active components like pentacyclic triterpenoids and flavonoids, among which polysaccharides are the main and most abundant functional components [4]. Polysaccharides, as important biological macromolecules widely present in plants, animals, and microorganisms [5], exhibit various pharmacological activities in *R. laevigata* Michx., including antioxidant, anti-inflammatory, hypolipidemic, liver protection, antibacterial, immunomodulatory, antitumor, and hypoglycemic effects [6]. Due to their ideal functional properties and rich resource reserves [4,7], the extraction of *R. laevigata* Michx. polysaccharides have attracted significant research interest.

Current mainstream extraction methods for plant polysaccharides include hot water extraction (HWE), microwave-assisted extraction (MAE), ultrasound-assisted extraction (UAE), acid extraction (FTACP), alkali extraction (FTAIP), and enzyme-assisted extraction (EAE) [8]. Among these, UAE has garnered significant attention in polysaccharide extraction due to its environmentally friendly nature, high efficiency, and low energy consumption. This technique leverages ultrasonic cavitation, mechanical vibration, and thermal effects to disrupt plant cell structures, facilitating polysaccharide release while minimizing the loss of bioactive components. For instance, Song et al. [9]. compared six extraction methods for soluble dietary fiber (SDF) from passion fruit peel and found that UAE yielded products with the lowest oil absorption capacity, likely attributable to their smoother and more compact surface structure. Chen et al. [10] employed five methods (HWE, accelerated solvent extraction (ASE), UAE, MAE, and EAE) to extract polysaccharides from bamboo shoots (*Chimonobambusa quadrangularis*, CPS), revealing that CPS obtained through different methods exhibited distinct advantages, with UAE-extracted CPS demonstrating superior antioxidant activity. Geng et al. [11] compared the effects of six common methods for extracting polysaccharides from *Clitocybe squamulosa* (CSFPs), showing significant differences among extraction methods and suggesting that the suitable method should be selected based on the application requirements of CSFPs. Shi et al. [12] extracted polysaccharides from *Cornus officinalis* Sieb. et Zucc. using six methods (hot water extraction, microwave-assisted extraction, ultrasonic-assisted extraction, acid extraction, alkaline extraction, and composite enzyme extraction), finding that the structure, antioxidant activity, and physicochemical properties of polysaccharides varied with different methods, while their thermodynamic characteristics showed similar trends, indicating that targeted development can be conducted based on the characteristics of polysaccharides extracted by different methods. Although systematic research has been performed on polysaccharide extraction methods and their physicochemical properties [13], studies on the sample differences in *R. laevigata* Michx. polysaccharides extracted by different methods and the specific comparisons of their physicochemical properties with traditional HWE remain scarce.

Given the rich resources, high medicinal value, and extensive application of *R. laevigata* Michx. in traditional Chinese medicine [14,15], developing efficient extraction processes is crucial to avoid resource waste and enhance its utilization value, thereby promoting industrial development. Therefore, this study employs *R. laevigata* Michx. as the raw material to systematically compare the effects of UAE and five other methods on polysaccharide yield, physicochemical properties, cholesterol-binding capacity, and antioxidant activity. The findings aim to provide a theoretical foundation for the efficient extraction and application of *R. laevigata* Michx. polysaccharides.

## 2. Materials and Methods

### 2.1. Materials and Reagents

*R. laevigata* Michx. was purchased from a local market in Guilin, Guangxi Zhuang Autonomous Region, China, dried at 50 °C, crushed and sieved (40-mesh) and stored in a desiccator, and kept sealed for subsequent use.

Papain (Nanning Pangbo Biotechnology Engineering Co., Ltd., Nanning, China); anhydrous ethanol (Beijing Aoboxing Biotechnology Co., Ltd., Beijing, China); hydrochloric acid (Chongqing Changpeng Chemical Co., Ltd., Chongqing, China); 1,1-diphenyl-2-picrylhydrazyl (DPPH, Shanghai Aladdin Biochemical Technology Co., Ltd.,Shanghai, China); phenol (Chengdu Jinshan Chemical Reagent Co., Ltd., Chengdu, China); anhydrous sodium acetate solution (Guangdong Guanghua Sci-Tech Co., Ltd., Guangzhou, China); glacial acetic acid (Chengdu Jinshan Chemical Reagent Co., Ltd., Chengdu, China); 2,4,6-tripyridyltriazine (TPTZ), 2,2′-azino-bis-(3-ethylbenzothiazoline-6-sulfonic acid) (ABTS, Shanghai Yuanye Biotechnology Co., Ltd., Shanghai, China); FeCl_3_ solution, ammonium ferric sulfate, petroleum ether, sodium hydroxide, cellulase (Sinopharm Chemical Reagent Co., Ltd., Shanghai, China).

### 2.2. Material Preparation

Before extraction, *R. laevigata* Michx. powder was degreased with petroleum ether (1:20, g:mL) (high-speed multifunctional grinder: HC-800Y, Yongkang Tianqi Shengshi Industry & Trade Co., Ltd., Jinhua, China). Subsequently, 100 g of the degreased crushed powder was weighed and added with distilled water at a solid-to-liquid ratio of 1:30 (g:mL) to prepare the extraction mixture.

Next, the polysaccharide extracts obtained by the six methods were centrifuged to remove impurities (benchtop high-speed centrifuge: H1650, Hunan Xiangyi Laboratory Instrument Development Co., Ltd., Changsha, China). Then, four volumes of anhydrous ethanol were added to the concentrated polysaccharide solution for alcohol (final concentration 80%) precipitation, and *R. laevigata* Michx. polysaccharides were obtained by centrifugation. The obtained polysaccharides were then deproteinized using the Sevage method (repeated 3 times with a chloroform/butanol mixture, *v*/*v* 4:1) until no protein layer was visible. Finally, the aqueous phase was collected and freeze-dried (16-0362, Linhai Yonghao Vacuum Equipment Co., Ltd., Linhai, China) to obtain the purified polysaccharides [16].

### 2.3. Extraction Method of Polysaccharide from R. laevigata Michx

#### 2.3.1. Hot Water Extraction (HAE)

The method of Zhan et al. [17] was referred to with slight modification. The mixture prepared in Section 2.2 was heated under reflux in a water bath at 80 °C for 2 h. After extraction, the solution was concentrated via evaporation using a rotary evaporator (RE-5298A; Shanghai Yarong Biochemical Instrument Factory, Shanghai, China). The extract was collected to obtain the hot water extract of *R. laevigata* Michx. polysaccharides.

#### 2.3.2. Microwave-Assisted Extraction (MAE)

The method of Rostami, Hosein et al. [18] was referenced with appropriate modification. The mixture prepared in Section 2.2 was heated under reflux in a microwave oven at a fixed power of 438 W for 3.5 min, and this heating cycle was repeated three times. The extract was collected to obtain the microwave-assisted extract of *R. laevigata* Michx. polysaccharides.

#### 2.3.3. Ultrasound-Assisted Extraction (UAE)

The method of Jooyandeh, Hossein et al. [19] was referred to with slight adjustment. The mixture prepared in Section 2.2 was heated under reflux in a water bath at 80 °C for 20 min, followed by ultrasonic treatment for 20 min using an ultrasonic cleaner (SB-120D, Ningbo Xinzhi Biotechnology Co., Ltd., Ningbo, China), with three repeated cycles. The extract was collected to obtain the ultrasound-assisted extract of *R. laevigata* Michx. polysaccharides.

#### 2.3.4. Acid Extraction (FTACP)

This method was modified slightly based on the protocol by Shi et al. [13]. Briefly, 100 g of *R. laevigata* Michx. powder was weighed and mixed with 0.06 mol/L HCl solution at a solid-to-liquid ratio of 1:30 (g:mL). The mixture was heated under reflux in a water bath at 80 °C for 2 h, after which the residue was removed by filtration. The pH of the resulting filtrate was adjusted to neutrality using sodium hydroxide (NaOH) solution, and the pH value was monitored in real time with a pH meter (247,258; Shanghai Ridao Scientific Instrument Co., Ltd., Shanghai, China). The extract was collected to obtain the acid extract of *R. laevigata* Michx. polysaccharides.

#### 2.3.5. Alkali Extraction (FTAIP)

Similarly, this method was adjusted slightly with reference to the study by Shi et al. [13]. A total of 100 g of *R. laevigata* Michx. powder was weighed, mixed with 0.2 mol/L NaOH solution at a solid-to-liquid ratio of 1:30 (g:mL), and heated under reflux in a water bath at 80 °C for 2 h, followed by suction filtration to separate and remove the insoluble residue. Subsequently, the pH of the filtrate was adjusted to neutrality using hydrochloric acid (HCl) solution. The extract was collected to obtain the alkali extract of *R. laevigata* Michx. polysaccharides.

#### 2.3.6. Enzyme-Assisted Extraction (EAE)

The method of Song et al. [20] was referenced with appropriate modification. The extraction mixture prepared in Section 2.2 was supplemented with 4% papain and cellulase (papain:cellulase = 3:1) for enzymatic hydrolysis, and then heated under reflux in a water bath at 80 °C for 2.0 h. The extract was collected to obtain the enzyme-assisted extract of *R. laevigata* Michx. polysaccharides.

### 2.4. Determination of Polysaccharide Yield

The phenol–sulfuric acid method [17] was adopted. Glucose was prepared into solutions with different concentration gradients. Then, 2 mL of each glucose solution was precisely measured, added with 5 mL concentrated sulfuric acid and 1 mL 5% phenol solution, kept at room temperature for 20 min, and the absorbance was measured at 490 nm. A standard curve was plotted with glucose concentration as the abscissa and absorbance as the ordinate.

A total of 100 mg of *R. laevigata* Michx. polysaccharides extracted by different methods was accurately weighed, dissolved in a 200 mL volumetric flask, and made up to volume with distilled water. The absorbance was measured according to the above steps, and the polysaccharide yield was calculated by substituting into the standard curve.

### 2.5. Determination of Physicochemical Indices

#### 2.5.1. Oil Absorption Capacity

The method of Song et al. [9] was referred to with slight modification. 0.10 g of *R. laevigata* Michx. polysaccharides extracted by different methods were accurately weighed and placed in a centrifuge tube containing 5 mL salad oil (food-grade rapeseed oil, a local brand), shaken for 1 min to completely immerse the polysaccharides in salad oil, and then centrifuged at 4000 r/min for 10 min to measure the volume of free oil. The oil absorption capacity of *R. laevigata* Michx. polysaccharides was calculated as follows:

(1) Oil Absorption Capacity = (V_1_ − V_2_)/m

V_1_: volume of added salad oil (mL), V_2_: volume of free oil after reaction (mL), m: mass of polysaccharides (g).

#### 2.5.2. Foaming Capacity and Foam Stability

A total of 0.30 g of *R. laevigat* polysaccharides extracted by different methods was accurately weighed and dissolved in 100 mL distilled water. The solution was stirred with a high-speed homogenizer at 2000 r/min for 3 min, quickly poured into a graduated cylinder, and the foam volume (V_0_) was recorded for foaming capacity determination. After standing for 30 min, the foam volume (V_30_) was recorded again for foam stability determination [21]. The calculations were as follows:

(2) Foaming Capacity (%) = V_0_/V_solution_ × 100%

(3) Foam Stability (%) = V_30_/V_0_ × 100%

V_0_: foam volume after stirring (mL), V_30_: foam volume after standing for 30 min (mL), V_solution_: total volume of polysaccharide solution (mL).

### 2.6. Determination of Cholesterol Adsorption Capacity

The cholesterol content was determined with modifications based on GB/T 5009.128-2016 standard [22]. Absorbance was measured at 564 nm to plot the standard curve.

The in vitro cholesterol adsorption assay of *R. laevigata* Michx. polysaccharides was optimized according to the reference [23]. A total of 200 mg of polysaccharides extracted by different methods was accurately weighed and dissolved in 5 mL distilled water. Then, 0.5 mL of the 20 mg/mL polysaccharide solution was precisely measured and mixed with 2.5 mL cholesterol solution (2.5 mg/mL), followed by shaking in a water bath (37 °C) for 100 min and centrifugation for 10 min. A total of 2 mL of the supernatant was transferred to a centrifuge tube containing 3 mL distilled water, added with 0.7 g sodium chloride and 10 mL petroleum ether, shaken for 2 min, and allowed to stand for 60 min for extraction. Then, 2 mL of the extract was mixed with 4 mL glacial acetic acid and 2 mL ferric alum color-developing solution, shaken well, and kept for 15 min before absorbance measurement at 564 nm. The cholesterol adsorption capacity was calculated as follows:

(4) Cholesterol Adsorption Capacity = ((C_0_ − C_1_) × V)/m

C_0_: initial cholesterol concentration (mg/mL), C_1_: cholesterol concentration after reaction (mg/mL), V: total volume of reaction system (mL), m: mass of polysaccharides (g).

### 2.7. Determination of Antioxidant Activity

#### 2.7.1. DPPH Radical Scavenging Capacity

A total of 0.0079 g of DPPH was weighed and dissolved in anhydrous ethanol to a final volume of 100 mL. A 20 μg/mL *R. laevigata* Michx. polysaccharide solution was prepared. Then, 50 μL of the solution was added to a 96-well plate, followed by 150 μL DPPH solution, and reacted in the dark for 30 min. Using distilled water as the blank control, absorbance was measured at 517 nm with three parallel replicates. The DPPH radical scavenging rate was calculated as

(5) DPPH Radical Scavenging Rate (%) = (1 − A_1_/A_0_) × 100%

A_0_: absorbance of the blank group, A_1_: absorbance of the sample group.

#### 2.7.2. ABTS Radical Scavenging Capacity

A total of 0.0384 g of ABTS and 0.0070 g of potassium persulfate were separately dissolved in 10 mL volumetric flasks, mixed at a 1:1 (*v*:*v*) ratio, and reacted in the dark for 12 h. The mixture was diluted 25–30 times with distilled water to adjust the absorbance to approximately 0.7 at 734 nm. 30 μL of the 20 μg/mL polysaccharide solution was added to a 96-well plate, followed by 170 μL ABTS working solution, and reacted in the dark for 30 min. Absorbance was measured at 734 nm using distilled water as the blank control, with three parallel replicates. The calculation formula for ABTS radical scavenging rate was identical to that of DPPH.

#### 2.7.3. Ferric Ion Reducing Antioxidant Power (FRAP)

The FRAP working solution (pH = 3.6, containing 0.3 mol/L acetate buffer, 10 mmol/L TPTZ, 20 mmol/L FeCl_3_) was prepared at a volume ratio of 10:1:1 (*v*:*v*:*v*). A total of 50 μL of FeSO_4_ solutions with different concentrations (0, 0.01, 0.02, 0.04, 0.06, 0.08, 0.09, 0.10 mmol/L) was mixed with 150 μL of the working solution, reacted at 37 °C for 10 min, and the absorbance was measured at 593 nm to plot the standard curve.

A 0.1 mg/mL *R. laevigata* Michx. polysaccharide solution was prepared, and its absorbance was measured following the above procedure. The FeSO_4_ equivalent (1 FRAP unit defined as the absorbance of 1 mmol/L FeSO_4_) was calculated by substituting the sample absorbance into the standard curve, with three parallel replicates.

### 2.8. Determination of Structural Characterization

#### 2.8.1. Fourier Transform Infrared Spectroscopy (FT-IR)

The FT-IR spectra of *R. laevigata* Michx. polysaccharides were determined using a spectrometer (Nicolet iZ-10, Thermo Fisher Scientific, Waltham, MA, USA). The 1.20 mg polysaccharide powder extracted by different methods was accurately weighed and mixed with 150 mg dried KBr for tabletting. FT-IR analysis was performed in the wave number range of 4000–400 cm^−1^.

#### 2.8.2. Scanning Electron Microscopy (SEM)

A scanning electron microscope (HITACHI Regulus 8100, Hitachi, Tokyo, Japan) was used to observe the particle and microstructure of *R. laevigata* Michx. polysaccharides at an accelerated voltage of 10 kV. Small amounts of polysaccharide samples were placed on the sample stage, sputter-coated with gold, and observed using SEM.

#### 2.8.3. Thermogravimetric Analysis (TGA)

A total of 6.1403 mg of *R. laevigata* Michx. polysaccharides was accurately weighed and analyzed under a nitrogen atmosphere. The temperature was increased from 25 °C to 800 °C at a rate of 10 °C/min using a thermogravimetric analyzer.

#### 2.8.4. X-Ray Diffraction (XRD) Analysis

The crystal structure of polysaccharides was characterized by XRD. The measurement conditions were set as follows: CuKα radiation, tube voltage 40 kV, tube current 40 mA, scanning angle range 5–80° (2θ), and step size 0.02°.

### 2.9. Statistical Analysis

The results were expressed as mean ± standard deviation (*n* ≥ 3). One-way analysis of variance (ANOVA) and Duncan’s multiple range tests in SPSS 27.0 software (IBM, Chicago, IL, USA) were used to analyze the significant differences (*p* < 0.05). GraphPad Prism 9.5.1 (GraphPad Software, San Diego, CA, USA) and Excel 2019 software were used for data analysis and graph processing

## 3. Results and Discussion

### 3.1. Analysis of Polysaccharide Yield

**Figure 1:**
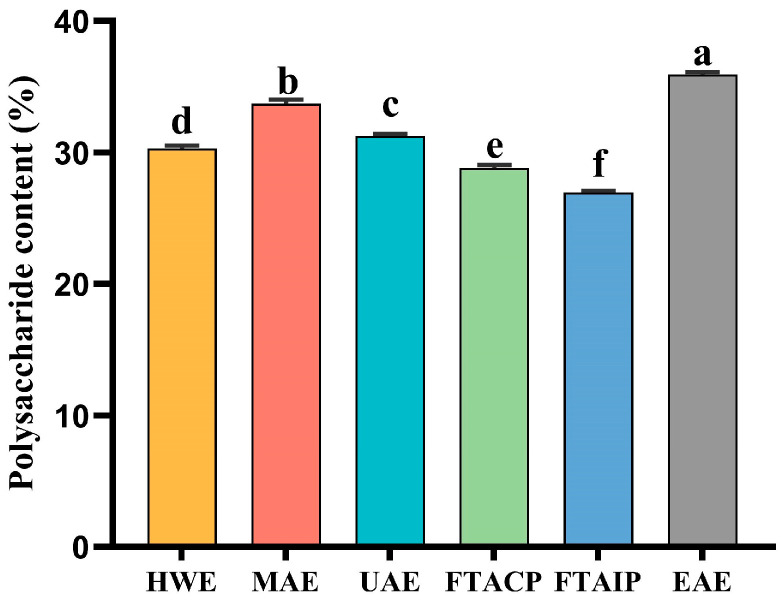
The yield of *R. laevigata* Michx. polysaccharide extracted by different methods. (*n* = 3; different lowercase letters between each group indicate significant differences, *p* < 0.05).

As shown in Figure 1, significant differences (*p* < 0.05) were observed in the polysaccharide yields obtained by the six extraction methods. Among them, the UAE yield was 31.27%, which was approximately 1.0%, 2.5%, and 4.3% higher than those of HWE (30.29%), FTACP (28.81%), and FTAIP (26.95%), respectively. However, it was about 4% lower than the yields obtained by EAE (35.95%) and MAE (33.70%).

This trend aligns with previous findings on *Ziziphus jujuba* Mill. Polysaccharides [24], suggesting that ultrasonic cavitation effectively disrupts the cellular structure of *R. laevigata* Michx., thereby enhancing polysaccharide release. Although EAE exhibited the highest yield—likely due to the enzymatic degradation of cell walls [25]—its potential for large-scale application may be influenced by the relatively high cost of enzymes and the need for precise control of reaction conditions, which warrants further economic evaluation. The superior yield of MAE can be attributed to microwave-induced thermal effects, which accelerate polysaccharide dissolution. In contrast, the lower yields of FTACP and FTAIP may result from unsuitable pH conditions, which could cause glycosidic bond cleavage and polysaccharide degradation [26]. Therefore, a careful balance between extraction efficiency and polysaccharide stability should be considered in practical applications.

### 3.2. Analysis of Physicochemical Indices

#### 3.2.1. Analysis of Oil Absorption Capacity

**Figure 2:**
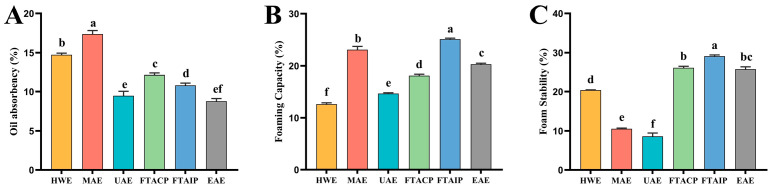
Comparison of physicochemical indices of *R. laevigata* Michx. polysaccharide extracted by different methods. (**A**) Oil absorbency; (**B**) Foaming capacity; (**C**) Foam stability. (*n* = 3; different lowercase letters between each group indicate significant differences, *p* < 0.05).

The oil absorption capacity is a critical physicochemical property of polysaccharides, primarily determined by their hydrophobic characteristics and molecular structure. As illustrated in Figure 2A, the UAE-extracted *R. laevigata* polysaccharides exhibited an oil absorption capacity of 9.47%, which was lower than that of MAE (17.37%) but significantly higher than EAE (8.77%). This phenomenon may be attributed to ultrasonic treatment inducing moderate molecular extension of polysaccharides, thereby exposing more hydrophobic groups and enhancing their affinity for oils. The superior oil absorption capacity of MAE-derived polysaccharides could be associated with microwave-induced structural reorganization of hydrophobic domains [12], while HWE (14.73%) maintained a favorable oil absorption capacity due to the preservation of intact hydrophobic regions under mild extraction conditions. In contrast, the inferior performance of EAE-extracted polysaccharides might result from enzymatic hydrolysis damaging hydrophobic domains or causing excessive molecular fragmentation. The discrepancy with Xiong et al.’s findings [27] may be explained by interspecies variations in plant materials.

#### 3.2.2. Analysis of Foaming Capacity and Foam Stability

Foaming capacity reflects a polysaccharide’s ability to reduce surface tension, while foam stability indicates its capacity to maintain foam structure. While the foaming capacity (14.63%) and foam stability (8.62%) of UAE-extracted polysaccharides were not the most prominent among the six methods, their unique extraction mechanism holds significant research value. UAE achieves rapid extraction while preserving bioactivity through cavitation effects that efficiently disrupt plant cell walls [24], making it particularly valuable for preparing thermolabile polysaccharides. In contrast, although FTAIP demonstrated optimal foaming capacity (25.10%) and foam stability (29.11%), the strong alkaline conditions may induce polysaccharide degradation [28]. HWE showed the poorest foaming performance due to its mild extraction conditions being insufficient for effective cell wall disruption [10]. Notably, the inferior foam stability of UAE may be attributed to mechanical shear forces damaging polysaccharide spatial structures during ultrasonication [29], which provides clear direction for future optimization of ultrasonic parameters (e.g., power, duration) to improve foam performance.

### 3.3. Analysis of Cholesterol Adsorption Capacity

**Figure 3:**
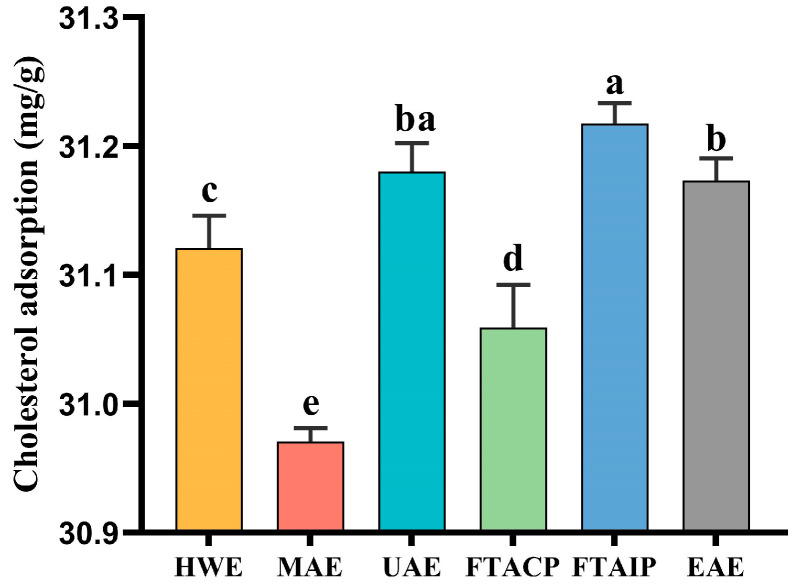
Cholesterol adsorption capacity of *R. laevigata* Michx. polysaccharide extracted by different methods. (*n* = 3; different lowercase letters between each group indicate significant differences, *p* < 0.05).

The UAE-extracted *R. laevigata* polysaccharides demonstrated remarkable cholesterol-binding capacity (31.18 mg/g), surpassing HWE (31.12 mg/g), MAE (30.97 mg/g), FTACP (31.06 mg/g), and EAE (31.17 mg/g) (Figure 3). Although it was merely 0.1% lower than FTAIP (31.22 mg/g), UAE achieves this comparable performance without the drawbacks of using harsh alkaline chemicals, highlighting its practical and environmental appeal. These results substantiate that ultrasonic treatment effectively activates polysaccharide molecules, where cavitation-induced mechanical forces promote molecular extension to expose more functional groups (e.g., hydroxyl and carboxyl groups) [30], thereby enhancing hydrogen bonding and hydrophobic interactions with cholesterol molecules. It is noteworthy that while alkaline conditions in FTAIP may induce polysaccharide chain unwinding [31], the strong alkaline environment could concurrently degrade some active groups and require additional neutralization steps. In contrast, as a green extraction technology, UAE maintains high adsorption performance without using harsh chemicals, making it more promising for functional food applications.

### 3.4. Analysis of Antioxidant Activity

**Figure 4:**
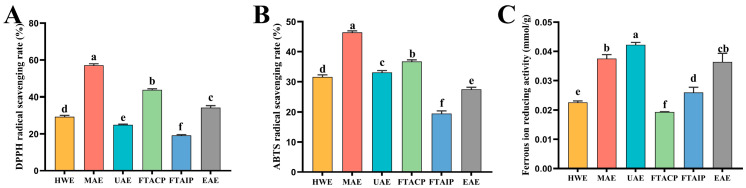
Comparison of antioxidant activity of different extraction methods. (**A**) DPPH free radical scavenging ability; (**B**) ABTS free radical scavenging ability; (**C**) Ferrous ion reducing activity. (*n* = 3; different lowercase letters between each group indicate significant differences, *p* < 0.05).

Polysaccharides with remarkable antioxidant activity play a crucial role in the functional food industry. By inhibiting lipid peroxidation and free radical chain reactions, they can effectively delay the oxidative degradation of nutritional components and maintain food quality and nutritional value [32]. This study systematically compared the antioxidant characteristics of *R. laevigata* polysaccharides obtained by different extraction processes using three complementary evaluation methods: DPPH radical scavenging capacity, ABTS radical scavenging capacity, and FRAP (Figure 4).

The results revealed that while UAE-extracted polysaccharides showed moderate performance in DPPH (24.93%) and ABTS (33.11%) radical scavenging tests, they demonstrated significant superiority in the FRAP assay (0.0423 mmol/g), which was 12.5% higher than the second-best method (MAE, 0.0376 mmol/g) and more than twice that of the poorest performer (FTACP, 0.0194 mmol/g). This notable advantage in reducing power underscores the unique capacity of UAE to preserve the redox-active groups within polysaccharides. This differential performance may indicate the uniqueness of UAE-extracted polysaccharides in antioxidant mechanisms: compared to direct free radical scavenging ability, they exhibit greater potential in inhibiting metal ion-mediated oxidation reactions (such as the Fenton reaction commonly found in food systems) [33]. These metal ion-catalyzed oxidation reactions are major contributors to nutrient loss during food processing/storage and oxidative stress damage in biological systems. Polysaccharides with high reducing power can effectively chelate Fe^3+^ and reduce it to less reactive Fe^2+^, thereby blocking the chain reaction formation of highly oxidative substances like hydroxyl radicals. It is noteworthy that although MAE showed optimal performance in direct radical scavenging tests (DPPH 57.12%, ABTS 46.41%), its high-temperature extraction conditions may lead to degradation of some heat-sensitive antioxidant components, a finding consistent with Zhao et al.’s report on the thermal stability of plant polysaccharides [34]. Furthermore, studies indicate that the antioxidant capacity of polysaccharides is closely related to their molecular weight and structural characteristics [35]. In contrast, the mild extraction conditions of UAE better preserve the integrity of redox-active groups (such as phenolic hydroxyl and enol structures) in polysaccharide molecules [36]. This characteristic endows UAE with unique advantages in developing functional foods that require maximum retention of natural antioxidant activity.

When viewed in the broader context of polysaccharides from medicinal plants, the UAE-extracted *R. laevigata* polysaccharides present a compelling functional profile. A comparative analysis with documented polysaccharides, such as those from sea buckthorn berries [28] and Radix *Bupleuri* [30], indicates that while the *R. laevigata* polysaccharides may not top every single metric, they exhibit a highly competitive and well-balanced combination of high FRAP reducing power and significant cholesterol-binding capacity. This balanced bioactivity profile, underpinned by the structural advantages (e.g., uniform porosity and higher crystallinity) preserved via the mild UAE process, positions *R. laevigata* polysaccharides as a promising multi-functional ingredient derived from a renewable resource for potential applications in functional foods.

### 3.5. Analysis of Structural Characterization

#### 3.5.1. Analysis of FT-IR

**Figure 5:**
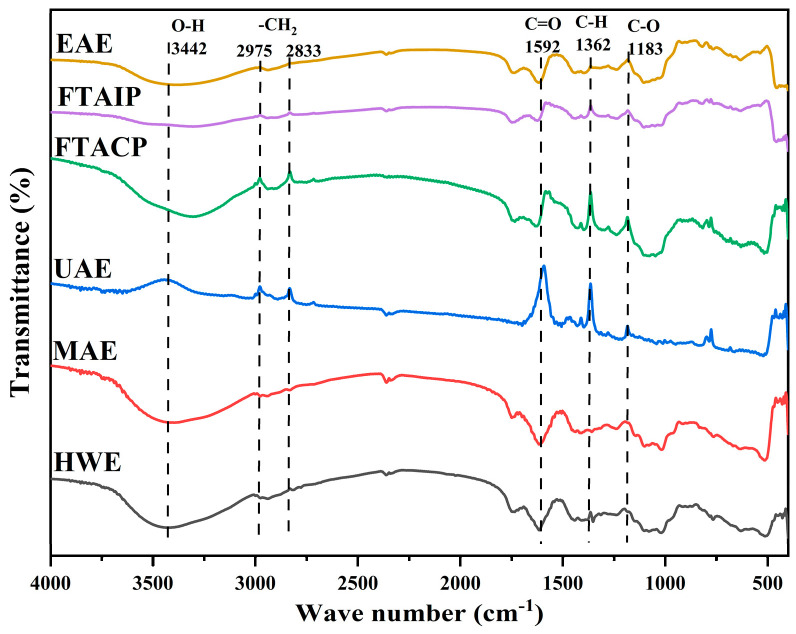
Infrared spectrum of polysaccharide extracted by different methods.

The FT-IR spectra of *R. laevigata* polysaccharides exhibited both common and distinct absorption peaks in the characteristic functional group regions (Figure 5), reflecting the fundamental chemical structural features of polysaccharides and the potential influence of extraction methods on their structures. Specifically, the broad peak near 3442 cm^−1^ was attributed to the O–H stretching vibration of polysaccharide molecules, indicating the presence of intermolecular or intramolecular hydrogen bonds. The absorption peaks at 2975 cm^−1^ and 2833 cm^−1^ corresponded to the –CH_2_ stretching vibration, confirming the existence of alkyl chain structures. The peak at 1592 cm^−1^ was associated with C=O stretching vibration, while the peaks near 1362 cm^−1^ and 1183 cm^−1^ originated from C–H bending vibration and C–O stretching vibration, respectively. These characteristic peaks collectively verified the fundamental carbohydrate skeleton of the polysaccharides [37].

Analysis of spectral peak shapes and intensities revealed that the polysaccharides obtained by UAE displayed sharper and more symmetrical absorption peaks for all characteristic functional groups (e.g., O–H, C–H, C=O, and C–O), with moderate peak intensities that distinctly differed from those of other extraction methods. This suggests that the UAE process caused minimal damage to the polysaccharide molecular structure, better preserving the integrity of their original functional groups. Specifically, the UAE-extracted polysaccharides showed reduced broadening of the O–H stretching vibration peak, indicating that the hydrogen bond network remained largely undisturbed during extraction, thereby helping to maintain the polysaccharides’ hydrophilicity, hydration capacity, and other physicochemical properties [38]. Moreover, the well-defined peaks corresponding to C=O and C–O vibrations, which reflect glycosidic bonds and ring structures, further demonstrated that UAE could efficiently extract *R. laevigata* polysaccharides with intact chemical structures. This advantage establishes a solid foundation for subsequent investigations into the bioactivities of these polysaccharides, such as immunomodulation and antioxidant effects.

#### 3.5.2. Analysis of SEM

**Figure 6:**
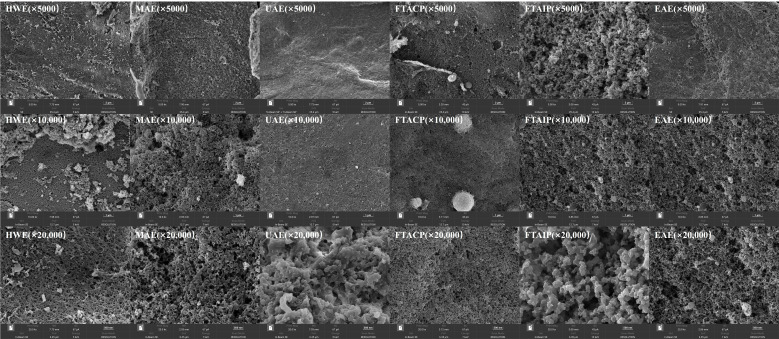
SEM image of polysaccharide extracted by different methods.

This study characterized the microscopic morphology of *R. laevigata* polysaccharides prepared by different extraction methods using SEM (Figure 6). At different magnification levels (5000×, 10,000×, and 20,000×), the polysaccharides obtained via UAE exhibited a uniform and fine particulate structure with a smooth surface and excellent dispersibility. Compared to other extraction methods, UAE employs ultrasonic cavitation to precisely disrupt cell walls while utilizing mechanical vibration to modulate intermolecular interactions, thereby promoting the ordered assembly of polysaccharide molecules [39]. This mild yet efficient extraction approach effectively preserves the native structure and bioactivity of polysaccharides. In contrast, HWE polysaccharides exhibited irregular agglomeration with rough surfaces and disordered pore distribution, which was consistent with previous findings by Zhao et al. [40]. Chemically extracted (FTACP/FTAIP) polysaccharides displayed rough surfaces and heterogeneous particle size distribution due to the influence of chemical reagents, resulting in loose molecular structures—a phenomenon consistent with previous reports [27]. Although MAE and EAE offer certain advantages, UAE demonstrates superior performance in terms of structural uniformity and surface characteristics.

#### 3.5.3. Analysis of TGA

**Figure 7:**
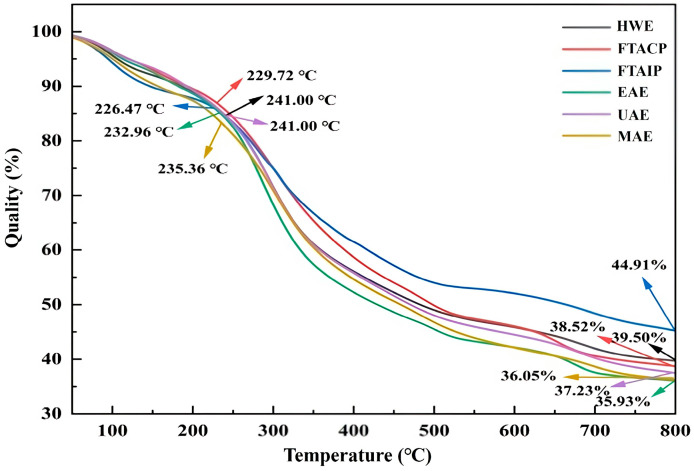
Thermogravimetric analysis of polysaccharide extracted by different methods.

Temperature variations can influence the morphology and structure of polysaccharides, thereby affecting their stability during storage and processing [41]. Therefore, this study employed TGA to analyze the thermal stability of *R. laevigata* polysaccharides extracted by different methods (Figure 7).

The TGA curves revealed that UAE exhibited superior thermal stability during the rapid weight-loss stage: its onset temperature of rapid degradation (241.00 °C) was higher than those of HWE (226.47 °C), FTACP (229.72 °C), and EAE (232.96 °C), indicating that UAE-extracted polysaccharides can maintain structural integrity at higher temperatures and resist thermal decomposition. This stage may be associated with the evaporation of free water in the samples [42].

Regarding residual mass, the UAE-extracted polysaccharides retained 37.23% at 800 °C, slightly lower than some methods (e.g., FTAIP at 44.91%). However, combined with the thermal degradation process, UAE polysaccharides exhibited a more gradual mass loss, suggesting minimal structural damage during extraction. This stage of mass loss may be attributed to polysaccharide depolymerization and thermal decomposition [43].

#### 3.5.4. Analysis of XRD

**Figure 8:**
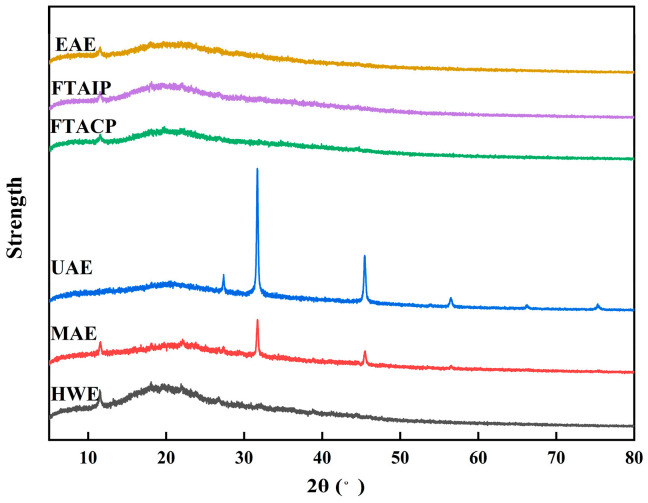
X-ray diffraction results of polysaccharide extracted by different methods.

XRD analysis revealed distinct differences in the number, position, and intensity of diffraction peaks among *R. laevigata* polysaccharides obtained by different extraction methods (Figure 8), reflecting the impact of extraction techniques on the molecular aggregation state and crystalline characteristics of polysaccharides [28]. Specifically: (1) HWE polysaccharides exhibited a flat baseline with diffuse and weak diffraction peaks, indicating low crystallinity and predominantly amorphous molecular arrangement; (2) MAE, FTACP, FTAIP, and EAE polysaccharides showed characteristic diffraction peaks, but with broadened peak shapes and limited intensity. This phenomenon may be attributed to molecular chain scission and crystalline region disruption caused by thermal effects, acid-base conditions, or enzymatic hydrolysis during extraction, thereby hindering the formation of well-defined crystal structures.

UAE polysaccharides, on the other hand, showed notable structural advantages: high-intensity, sharp diffraction peaks at 2θ = 30° and 45° indicated greater crystallinity and a more ordered crystalline structure. Such increased crystallinity has been shown in earlier research to considerably improve a number of polysaccharide physicochemical qualities, such as mechanical strength, viscosity, and ability to interact with biomolecules [44].

## 4. Conclusions

The effects of ultrasound-assisted extraction (UAE) on the extraction efficiency, physicochemical characteristics, and structural traits of *Rosa laevigata* Michx. polysaccharides were systematically compared with those of five conventional methods: hot water extraction (HWE), microwave-assisted extraction (MAE), acid extraction (FTAC), alkali extraction (FTAI), and enzyme-assisted extraction (AEE). According to the results, UAE performed better than HWE (30.29%), FTACP (28.81%), and FTAIP (26.95%) in several important indices. It also demonstrated the highest FRAP reducing power (0.0423 mmol/g) of any method and a remarkable cholesterol-binding capacity (31.18 mg/g), which was second only to FTAIP (31.22 mg/g). While FT-IR and TGA verified the UAE-extracted polysaccharides’ superior preservation of native conformation and thermal stability (initial decomposition temperature reached 241.00 °C), structural characterization (SEM, XRD) showed that they had a uniform porous network structure and higher crystallinity. In contrast to previous techniques, UAE effectively extracted polysaccharides while maximizing the preservation of their bioactivity by combining mechanical vibration with ultrasonic cavitation. These attributes, combined with its scalability and low environmental impact, position UAE as a viable technique for the industrial-scale production of *R. laevigata* polysaccharides for applications in dietary supplements and functional foods.

When considering the overall performance, UAE presents a more balanced and advantageous profile compared to MAE and EAE. While MAE and EAE achieved higher extraction yields, the potential for thermal degradation in MAE and the operational cost associated with EAE are notable drawbacks. UAE, in contrast, effectively balances a competitive yield with superior preservation of bioactivity (as evidenced by the highest FRAP value and remarkable cholesterol-binding capacity) and structural integrity, all while operating under milder, more environmentally friendly conditions. This combination of efficiency, bioactivity retention, and sustainability underscores the high-value potential of UAE for the industrial extraction of *R. laevigata* polysaccharides.

## Data Availability

The original contributions presented in this study are included in the article. Further inquiries can be directed to the corresponding authors.
